# Time and Generation: Parents’ Integration and Children’s School Performance in Sweden, 1989–2011

**DOI:** 10.1007/s10680-018-9498-9

**Published:** 2018-10-25

**Authors:** Christopher D. Smith, Jonas Helgertz, Kirk Scott

**Affiliations:** 1 Centre for Economic Demography, Lund University, Box 708, 220 07 Lund, Sweden; 2 Department of Economic History, Lund University, Box 708, 220 07 Lund, Sweden; 3 Institute for Social Research and Data Innovation and Minnesota Population Center, University of Minnesota, Minneapolis, MN USA

**Keywords:** Integration, Assimilation, Intermarriage, Family fixed effects, Intergenerational transmission

## Abstract

A central element of assimilation theory is that increasing time and number of previous immigrant generations in a host country leaves immigrants and their children more integrated and capable of navigating the host society. However, the underperformance of some immigrant groups in Sweden calls into question this relationship. Additionally, many studies regard intermarriage as an outcome of immigrant integration and rarely investigate whether integration continues after intermarriage. Using population level data from the Swedish interdisciplinary panel on 22 cohorts of ninth-grade students born between 1973 and 1995, we examine the effect of parents’ time in Sweden on their children’s grade point average using family fixed effects. Additionally, we investigate whether this relationship differs between “2.0” and “2.5” generation children. We find, generally, that parents’ time in Sweden increases their children’s educational performance, though some variation by parents’ region of origin exists. This supports the idea that integration experiences in immigrant families can be transmitted across generations. Further, this generally holds for both the 2.0 and 2.5 generation children. This relationship among the 2.5 generation is notable as previous studies using a family-based approach looking at the intergenerational transmission of integration have largely focused on the children of two foreign-born parents.

## Introduction

In the previous half-century, the proportion of the Swedish-born population with at least one foreign-born parent has risen from nearly 0 to 10% of the population. With a growing representation in the population, there is increasing interest in the outcomes of second-generation children in Sweden. Schooling has received a particular focus, with studies finding second-generation immigrants generally performing worse in school and attaining less education than children of native-born Swedes (Jonsson and Rudolphi 2011; Taguma et al. 2010). Although second-generation immigrants are, by definition, born in the host country, they represent a highly heterogeneous group, not only in terms of the parents’ countries of origin, but also in terms of the length of time their parents have resided in the host country. According to assimilation theory (Alba and Nee 2003; Gordon 1964), increasing time and number of previous immigrant generations in a given host society is—on average—expected to lead to improved integration for the descendants of individuals with foreign origins. However, with so many groups exhibiting poorer outcomes than natives, it is fair to ask why this is the case.^1^ This study assesses of second-generation immigrant integration, by examining the impact of parents’ integration, measured as parents’ years since migration, on their children’s academic performance. If parents’ integration experiences are transmitted across generations for some groups, but not others, this may contribute to the academic disadvantages observed among many children of immigrants.

Broadly, immigrants are expected to undergo a process of integration with time in a host country that broadly encompasses acculturation, linguistic development, socioeconomic structural assimilation and, possibly, intermarriage (Alba and Nee 2003; Bean and Stevens 2003). As a result, an increasing number of years spent in the host country should—on average—be positively associated with the amount of accumulated country-specific knowledge (National Academies of Sciences et al. 2016). This knowledge can subsequently be transmitted across generations to their children, possibly affecting their academic performance and educational attainment (Bleakley and Chin 2008; Turney and Kao 2009) as well as their social integration (Martinovic et al. 2009). Segmented assimilation theory, however, presents an alternative perspective that allows for multiple assimilation trajectories, and therefore a more complex relationship between time and number of previous generations spent in a country and integration (Portes 2006; Portes and Rumbaut 2001). Specifically, this link, as well as how it is transmitted across generations, might differ based on group characteristics and the context of reception experienced by different immigrant groups.

This study is among the first to investigate how parents’ time in the host country impacts the educational outcomes of second-generation immigrants, and how this differs by parental region of origin. This is done by studying the impact of parents’ years since migration on their children’s academic performance, and we use the Swedish interdisciplinary panel (SIP), which contains longitudinal register data until 2011 on all individuals born between 1973 and 1995 as well as their parents. As intermarriage is often studied as an outcome of integration (Lee and Bean 2010; Gordon 1964; Kalmijn and Van Tubergen 2010), we also differentiate between children with two foreign-born parents (2.0 generation) and those with one foreign and one native-born parent (2.5 generation). This distinction is made due to the differences that have been found between these groups on educational attainment (Kalmijn 2015). Additionally, by distinguishing the children of two foreign-born parents from children of intermarried parents, this study contributes to a small but burgeoning line of research looking at ongoing individual integration of intermarried immigrants (Dribe and Nystedt 2015; Furtado and Song 2015; Tegunimataka 2017), and expands on this to test for potential intergenerational effects.

## Theoretical Framework

### Assimilation Theories

Assimilation-based theories have long served as a cornerstone in guiding empirical research into how the foreign-born and their descendants integrate into their host societies. Although a distinction can be drawn between classic and new assimilation theory, both are centered around the idea that, all else being equal, the length of time an immigrant spends in a host country will have a positive impact on integration (Alba and Nee 2003; Gordon 1964). Drawing on new assimilation theory, integration is the result of day-to-day decisions that immigrants make to maximize their well-being (Alba and Nee 2003). Immigrants will, according to this theory, undertake pragmatic decisions to improve their quality of life, such as becoming more proficient in the local language, increased interaction with natives, familiarity with host country culture and norms, upward mobility through education or economic achievement as well as an increased understanding of the way institutions function (Brown and Bean 2006). Taken together, these are broadly defined as the processes of acculturation and structural assimilation. Though these actions may not be undertaken with the long-term intent of integrating, the by-product of these decisions is a general increase in the integration of immigrants. In becoming familiar with the host country culture as well as embedding oneself in the structural world of the host country, this provides opportunities for immigrants to cross the socially constructed boundaries that exist between minority and majority groups (Alba 2005, 2009). Within families with second-generation immigrant children, this may result in foreign-born parents being more comfortable and better equipped to become involved with their children’s education program the longer the time they have spent in the country (Turney and Kao 2009). Although the expectation is that all groups will derive benefits from time spent in the host country, immigrants represent a spectrum of backgrounds and experiences. Contexts like contemporary Sweden, with its considerable mix of immigrant groups, imply that the socioeconomic and cultural “distances” that individuals have to overcome to converge with the mainstream may be substantial depending on origin country (Dribe and Lundh 2011). Despite the potential variation in aforementioned “distances,” assimilation theory predicts a gradual processes of acculturation and structural assimilation, from which the first hypothesis of the paper is derived:

#### **H1**

Among children of foreign-born individual(s), the parents’ time in the host country positively influences children’s academic performance.

Conversely, the segmented assimilation hypothesis puts forth that, although time spent in the host country can lead to integration and upward social mobility for many groups, it is not preordained (Portes and Rumbaut 2001; Portes and Zhou 1993; Massey and Sanchez 2011). Instead, integration can occur along one of several trajectories, which are strongly influenced by individual and group socioeconomic and cultural characteristics. Many groups will follow the previously described normative assimilation path or through assimilation with retained biculturalism, both of which would predict a positive relationship between parents’ time spent in a host country and their integration. Alternatively, foreign-born individuals from groups who tend to be the most culturally and ethnically different, possessing low levels of human capital obtained in the country of origin and who experience discrimination from the native population, are at risk of undergoing a process of downward assimilation (Haller et al. 2011; Portes and Fernández-Kelly 2008). Drawing on the concept of boundary formation (Alba 2005, 2009), foreign-born individuals from these groups can be said to have a “bright” boundary that exists between them and the native population that makes the process of acculturation and structural assimilation more difficult to achieve. In such households, an uneven process of integration can result in parents integrating slower than their native-born children (Portes and Zhou 1993). This situation is likely to arise when migrants have children shortly after arrival, which is more typical of low-educated immigrants coming from non-Western countries of origin and can cause tensions in the household (Harker 2001). As a result, parents are linguistically and socially less comfortable than their children, even coming to rely on them as translators in formal settings, leading them to feel less able and/or comfortable in assisting their children in school (Portes and Zhou 1993; Portes and Rumbaut 2001). Furthermore, due to these parents having limited language skills, their children are generally less exposed to the host country language as infants and young children, limiting their linguistic development. Children may also view the limited mobility of their parents and their co-ethnic peers as evidence that success, as defined by the mainstream, is not available to them, resulting in a disinterest of education as a pathway for upward mobility (Haller et al. 2011; Simón et al. 2014). Additionally, groups who experience downward assimilation have fewer economic and cultural resources available for their second-generation children due to their increased risk of social isolation:

#### **H2**

Among children of foreign-born individuals, parents belonging to the most culturally and socioeconomically distant groups, at the greatest risk of experiencing downward assimilation, will display a neutral relationship between years since migration and their children’s academic performance.

### Intermarriage

Sociologists have long regarded aggregated rates of intermarriage as a measure of social distance between groups (Davis 1941; Merton 1941). Gordon (1964) subsequently extended this to the field of immigrant integration, where he posited that intermarriage only occurs after structural assimilation and acculturation has been achieved, thus marking an advanced stage of a minority group’s integration into the majority group culture. This perspective, that a prevalence of intermarriage signifies a decline in the salience of racial and ethnic boundaries and cultural distinctiveness, can also be found in contemporary studies on the topic (Lee and Bean 2010; Dribe and Lundh 2008; Huschek et al. 2012; National Academies of Sciences et al. 2016). Empirical research on contemporary Sweden suggests that such boundaries exist, as those groups who are the most culturally and ethnically similar to native Swedes display the highest rates of intermarriage (Dribe and Lundh 2011), as well as elsewhere in Europe (Kalter and Schroedter 2010; Muttarak and Heath 2010; van Tubergen and Maas 2007). Based on boundary formation theory (Alba 2005, 2009; Lichter et al. 2011), an interpretation of this would be that those who come from more similar countries of origin who practice the same religion have an easier time achieving this due to fewer social boundaries between the groups (Dribe and Lundh 2011; Kalmijn and Van Tubergen 2010). In Sweden, the extensive intermarriage patterns between natives and immigrants from Western countries with similar backgrounds may reflect individual preferences rather than societal limitations. Conversely, those groups with non-Western backgrounds have a “brighter,” or more accentuated, boundary with natives, and thus intermarriage more reflects an individual crossing of an intact boundary between groups. Consequently, intermarriage for individuals with a non-Western background may represent a very select subsample of the group.

Studies frequently treat intermarriage as an outcome of successful acculturation and structural assimilation (Dribe and Lundh 2008; Kalmijn 1998, 2012; Lichter et al. 2015; Lee and Bean 2010). However, questions persist whether this is due to selection into intermarriage (van Tubergen and Maas 2007; Muttarak and Heath 2010), in which intermarried parents tend to be positively selected on education (Kalmijn 2012), come from cultural similar countries of origin (Kalmijn and Van Tubergen 2010; Dribe and Lundh 2011; Kalter and Schroedter 2010) and have attained greater language proficiency (Becker 2011). This tends to result in children of intermarried couples outperforming those of two immigrant-origin parents (Kalmijn 2015; Ramakrishnan 2004). A shortcoming of a number of these studies is the difficulty to separate selection into intermarriage from the consequences of being married to a native partner or the child of their union (Song 2009). There is, however, a limited understanding regarding whether intermarriage bequeaths additional advantages or merely reflects already attained social status. Intermarriage as a vehicle for integration has, however, been receiving increased focus in recent years (Dribe and Nystedt 2015; Furtado and Song 2015; Tegunimataka 2017), but selection into intermarriage and the lack of appropriate data make this subject difficult to study. The studies that have focused on intermarriage as an event rather than as an outcome of integration have found that it increases the foreign-born individual’s social network (Goldstein 1999; Laumann et al. 1994) and sometimes also improves their labor market outcomes (Dribe and Nystedt 2015; Furtado and Song 2015; Tegunimataka 2017), with benefits also to their offspring (Kalmijn 2015). Thus, to the extent that the degree of social and economic integration transmits across generations and continues past the event itself, a different effect of parents’ years spent in the host country is expected between individuals whose foreign-born parent intermarries a native and those who have two foreign-born parents. These differences should, furthermore, be the most accentuated among groups characterized by the greatest socioeconomic and cultural distance to Sweden, where the act of intermarriage is expected to be associated with the largest gains (Dribe and Nystedt 2015). Furthermore, if the acquisition of social and economic capital continues throughout the duration of the intermarriage and the benefits carries over generations (Dribe and Nystedt 2015; Furtado and Song 2015; Elwert and Tegunimataka 2016; Goldstein 1999), the effect of years since migration for the foreign-born parent should also be positive for the 2.5 generation, although the underlying mechanisms may differ from that of the 2.0 generation. From this, we derive the following hypotheses:

#### **H3a**

The effect of parents’ years spent in the host country is greater among individuals whose foreign-born parent intermarries a native than those who have two foreign-born parents.

#### **H3b**

The effect of intermarriage increases with socioeconomic and cultural distance.

Despite the possible advantages of intermarriage among the parents of second-generation immigrant children, this perspective fails to take into account the possibility of stigmatization, particularly among the children with a parent who is a visible minority (Khanna 2010; Edwards et al. 2012). This may lead to the child being identified as a minority, thereby facing the associated discrimination and stigma experienced by other second-generation immigrant children, effectively limiting the benefit that may be derived from having mixed ancestry. While there are potentially lingering disadvantages experienced by the 2.5 generation, they enjoy several benefits that do not extend to the 2.0 generation. More specifically, the 2.5 generation grows up in a household with at least one fluent native speaker present, as well as the possibility for a more expansive network of natives, both linked to the native parent, and thus largely unrelated to the number of years, the foreign-born parent has resided in the host country. Previous research on the intergenerational relationship between parents’ years spent in a host country and child’s academic aptitude focuses on linguistic development and class performance (Bleakley and Chin 2008; Casey and Dustmann 2008; Smith et al. 2016), which should be less important in a household with one fluent speaking native and one positively selected foreign-born parent.

Alternatively, if intermarriage only occurs in light of complete or nearly complete integration for an individual and precedes childbirth, the benefits of additional time in Sweden should not provide additional integration benefits, suggesting no accentuation of the relationship between parents’ time in Sweden and children’s educational performance among the intermarried, across all groups. Furthermore, if the foreign-born parent’s years since migration primarily affects the children’s academic performance through linguistic development, as was found in previous research (Smith et al. 2016), the 2.0 generation should receive the greatest benefit from the parent(s) having been in the host country for longer compared to the 2.5 generation.

#### **H4**

If the primary destination country-specific benefits among children of intermarriage stem from the native-born parent, the effect of the foreign-born parent(s) years since migration is greater among 2.0 than among 2.5 generation children.

## Previous Research

Research looking at immigrant integration through an intergenerational lens is an emerging area of research (Kulu and González-Ferrer 2014). Similar to this study, those that have examined the intergenerational transmission of integration have frequently considered the effect of parents’ years since migration on some aspect of their children’s educational performance. As already outlined, this is motivated by the expectation that time spent in the host country roughly captures the accrued effects of structural integration and acculturation. Structural integration allows parents to provide their children with more resources while living and working in areas with more natives, which has been found to aid the integration of the children (Martinovic et al. 2009). Additionally, through the process of acculturation, parents would become better able to aid and assist their children’s educational performance. Examining the aforementioned mechanisms using an intergenerational approach, studies find that parents’ linguistic acculturation, as proxied with time spent in a host country, improves their child’s language skills and grades in language classes (Bleakley and Chin 2008; Casey and Dustmann 2008). By growing up in households in which the host country’s language would be more readily comprehended and spoken, children are provided additional linguistic aptitude that should allow them to perform better in school. Parental language acquisition and child’s proficiency represent direct effects of how parents’ integration may influence their child’s educational performance and attainment (Kristen 2008; Kristen et al. 2011). More broadly, parental integration could lead to increased institutional familiarity and a reduced negative impact of cultural distance on children’s school performance (Turney and Kao 2009; Kristen 2008) as well as increased social and cultural capital (Bourdieu 1986; Lareau 2011) and the parent viewing migration as a permanent rather than a temporary move (Dustmann 2008). As a result, both structural integration and acculturation are capable of independently positively influencing parents’ capacity for affecting their children’s educational performance. This is expected to lead to increased engagement, which has shown to positively influence the educational performance of children of immigrants (Liu and White 2017).

A frequent limitation of integration studies is that they suffer from issues of selectivity and omitted-variable bias, in our case that the relationship between parents’ years since migration and children’s educational performance can be upwardly biased (Chiswick and Miller 1995), necessitating alternative strategies to estimate the effect. To this end, Åslund et al. (2009), Nielsen and Rangvid (2012), and Smith et al. (2016) employ family-based designs to examine the effect of parents’ number of years since migration on their children’s educational outcomes. Generally, these studies find that parents’ time spent in the country has a positive effect on their child’s educational performance, suggesting that although previous studies may have estimated upwardly biased links, they do have the correct sign. Åslund et al. (2009), using Swedish data, find that the child’s total years of education would be extended by 0.2 years if a parent had arrived a decade earlier, while Nielsen and Rangvid (2012) find that mother’s years since migration exercises a positive effect on the child’s performance in Danish, while father’s years since migration has a positive effect on their math performance. Smith et al. (2016), looking across a broader range of immigrant groups, find a generally positive effect of parents’ years since migration on their children’s Swedish performance, but no effect on math performance is observed.

## Swedish Context

Although Sweden has a relatively short immigration history, it has seen its share of first- and second-generation immigrants rise considerably in recent decades, today surpassing the USA in terms of its share of first-generation immigrants. Though this time period has been characterized by a rather steady inflow of foreign-born individuals, its composition has changed dramatically (Bengtsson et al. 2005; Westin 2003). After World War II, Sweden experienced a manufacturing and industrial boom from increased demand during the reconstruction of war-torn European countries. To meet labor demands, Sweden began actively recruiting foreign workers. This initially consisted of labor migration from other Nordic countries and eventually expanded to include other European countries, notably Germany, Italy, Austria, the former Yugoslavia, as well as Turkey. Following an economic slowdown in the early 1970s and changes in immigration policy, labor migration came to a virtual halt (Bengtsson et al. 2005; Van Mol and de Valk 2016). However, shortly thereafter, Sweden began receiving increasing numbers of refugees along with their related and tied family members, first from Chile and other South American countries in the mid-1970s, then from the Middle East (Iran, Lebanon, Turkey and Iraq) in the 1980s and Eastern Europe (former Yugoslavia and the Soviet Union) as well as East African countries in the 1990s. As a result, over the last half-century, the proportion of first- and second-generation immigrants in Sweden has risen from virtually nonexistent to representing approximately a quarter of the Swedish population and shifted from predominantly European to non-Western in origin.

Today, Sweden has a fairly diverse immigration experience. According to the Swedish Migration Board (2017), among those receiving residence permits in 2015, the largest category was family reunification with 40% of all immigrants, of which 15% were relatives of refugees and 14% were relatives of other Swedish residents. The second largest category was refugees who make up 33%. Of the remaining migrants, 16% were labor migrants, 3% were from EES states, and 9% were students. Nearly half of all immigrants to Sweden in 2015 were either refugees themselves or related to an earlier-arriving refugee. Additionally, migrants from other European Union countries amount to a number that is roughly on par with the inflow of labor migrants. This implies that refugees and their families are by far the largest single category of migrants, with roughly 85,000 individuals obtaining the right to remain in 2016.

The integration experiences of labor and humanitarian immigrants have differed markedly. Although the labor immigrants of the 1960s were largely unskilled, they had higher employment rates than natives and their children have achieved similar levels of educational attainment as natives (see Rosholm et al. 2006). As a result, these groups are generally labeled as successfully integrating into Swedish society.

In contrast, the integration of more recent, largely non-white and non-Western groups has been more difficult, with the first-generation immigrants experiencing worse labor market outcomes, such as lower employment rates (OECD 2015; Rosholm et al. 2006), and their offspring being characterized by worse educational performance and outcomes (Jonsson and Rudolphi 2011; Taguma et al. 2010). Over this time period, residential segregation along the lines of country of birth and socioeconomic status rose considerably, something which became particularly accentuated for non-white minority groups during the economic recession of the 1990s (Andersson 2007; Englund 1999). Additionally, during this economic crisis, employment rates among immigrants and their descendants, specifically those from non-Western countries of origin, declined considerably and have remained at levels far below native-born Swedes (Rosholm et al. 2006; Bevelander 2011; OECD 2016). Although some refugee groups arrived with high levels of human capital, notably Iranians and Chileans, most refugee groups are characterized as having large shares of individuals with low levels of education (Westin 2003). Despite their parents’ relatively high educational backgrounds, however, children with a Chilean or South American background have also faced difficulties in the Swedish labor market (Bengtsson et al. 2005; Rosholm et al. 2006; Scott 1999). While earlier-arriving labor migrant groups arrived with relatively low levels of education, they typically faced favorable labor market conditions. The same cannot be said for the later-arriving groups, entering a country that was undergoing a process of deindustrialization as well as a labor market placing high demands on formal education and informal skills, such as language (Rosholm et al. 2006; Scott 1999). This context parallels that of the USA for post-1965 immigrant groups who have had difficult integration experiences themselves (Portes and Rumbaut 2001; Portes and Zhou 1993). Further, many non-Western immigrants have come from non-Christian countries, and religion contributes greatly to the “bright” boundary between many of these immigrant groups and natives, even in largely secular Western Europe (Alba 2005). This combination of low human capital, few opportunities, segregation and hostile context of reception possibly leaves those arriving from the Middle East (with the exception of the previously mentioned Iranians), Asia and South America at risk of the aforementioned downward assimilation trajectory.

In terms of the context of reception, it is important to understand the social structures which are also important to integration, namely childcare and public schools. A unique characteristic about Sweden is the widespread availability of relatively inexpensive childcare from when the child is 1-year-old. This represents an alternative way for children of foreign-born individuals to become exposed to Swedish language and culture from outside the home environment. For this study, this could be problematic, since it is assumed that parents’ time in Sweden is a relevant proxy for such exposure. Nationally, about 80% of children under age 6 who are eligible enroll in some form of childcare (see Engdahl 2004). These rates are not available for immigrant groups or even by immigration status, however, and considering the low female employment rate and traditional gender roles for non-Western immigrant groups in particular, it becomes very difficult to assess the enrollment practices of these groups. Additionally, since residential segregation is common among non-Western immigrants (Andersson 2007), it is uncertain what the true exposure to Swedish language and culture in many of these child care institutions actually is.

The Swedish educational system is relatively open in terms of individual choice (Baysu and de Valk 2012; Jonsson and Rudolphi 2011), and grades only begin to formally matter for placement purposes during the transition from ninth grade to secondary school. So, unlike other systems that begin specialization at much earlier ages, Sweden delays making decisions with tangible consequences for the individual’s subsequent education until later. While this remains true to this day, a series of reforms were passed throughout the 1990s, leading to a greater decentralization of the school system. One reform in particular, the assessment reform of 1998, produced a notable surge in grade inflation that, however, was not uniformly distributed across student groups; ethnic Swedes in wealthier areas tended to experience a disproportionately large increase, while immigrants in poorer neighborhoods benefited less (Wikström 2005).

## Data and Methods

The data analyzed come from the Swedish Interdisciplinary Panel (SIP), administered at the Centre for Economic Demography. SIP contains longitudinal data on the entire Swedish population born between 1973 and 1995, as well as their parents and siblings born outside the main sampling window. Information on attained primary school grades is available from 1989 to 2011 and therefore constitutes the key period of interest. SIP also has information on demographic characteristics, including high-quality migration information (Aradhya et al. 2017), which provides migration history and region of birth. Through the addition of the multigenerational register, family identifiers have been created through linking parents to their biological children. This represents a particular strength of this study as a means of isolating the effects of integration and dealing with unobserved family heterogeneity (Lawlor and Mishra 2009; Angrist and Pischke 2008). Our study sample has been designed to consist of all children with at least one migrant parent, born in Sweden between 1973 and 1995. Furthermore, the individual must have reported a grade point average for ninth grade other than missing or incomplete. Lastly, this information must also be available for at least one other sibling, in order for family fixed effect models to be estimated, explained in more detail in Sect. 3.2.

### Measures

#### Educational Performance

The educational performance considered in this paper is represented by the ninth-grade grade point average for the years 1989 until 2011. This is the final grade of compulsory school and is of substantial importance as it determines both eligibility and admission to high school (*gymnasieskola*) in the following academic year. Around 90% of students continue to high school, where the options are vocational and academically oriented tracks, where the former prepares the student for manual work such as auto mechanic or electrician, whereas the latter prepares the student for subsequent academic studies at the tertiary level.

While minor changes to the compulsory school curriculum occurred over the time period examined, the student’s grade was throughout determined by a combination of the student’s performance in class and tests administered by the teacher. The grading of certain core subjects, namely Swedish, English, math and science (biology, physics, chemistry),^2^ were furthermore facilitated by national standardized tests, where the student’s performance is compared to a national average. The only major change that occurred concerned the grading practice which, until 1998, followed a relative integer scales from 1 (lowest) to 5 (highest) with the overall aim of grading which for the country as a whole followed a normal distribution. With time, concerns around the fairness that the grading system was more informative about the student’s performance compared to his or her class mates than about their actual knowledge of the subject lead to a change to an absolute scale from the academic year 1998, with three passing grades [pass (10), pass with distinction (15) and pass with high distinction (20)]. Both before and after the change in the grading system, the student’s final grade was calculated as a mean, and in the analysis, we standardize this grade by year (mean 0, standard deviation 1), thereby expressing performance relative to the individual’s graduating cohort. All models include a control for the post-1998 period, and sensitivity analyses have been conducted on the pre- and post-reform period, yielding virtually identical results as when analyzing the entire period.

Grade point average as an outcome is not without some disadvantage, the biggest of which is grade does not reflect only absolute cognitive skill and subject mastery. Instead, they also capture other factors that are known to influence grades, such as classroom behavior, student cultural capital, individual student characteristics and personality, and teacher differences in grading routines (Lounsbury et al. 2003; Kelly 2008; McGrady and Reynolds 2013; Lareau 2011; Covay and Carbonaro 2010). Standardized test, meanwhile, is thought to serve as a better measure of subject mastery and topic knowledge (Duckworth et al. 2012). However, international test, such as PISA, suffers their own disadvantages, such as low student motivation (Skolverket 2015), that may bias results. Despite the limitation of grade point average, it is preferred here due to its aforementioned importance in high school admission, which makes it more important to students than “low-stakes” standardized tests. Further, some of these non-cognitive factors may also be influenced by parents’ integration, which standardized test may fail to capture.

#### Parents’ Region of Origin

Our analysis will examine Swedish-born individuals with at least one foreign-born parent, examined through the following region of origin groups: Africa, Iraq, Iran, Lebanon/Turkey, Asia, South America, Eastern Europe, North America/EU-15^3^ and Oceania, and the Nordic countries.^4^ Each individual can only have one origin group, classified by the most precise definition (e.g., an individual born in Turkey belongs to the Lebanon/Turkey group, and not to the Asia group). The larger regional groupings were necessitated by a combination of the level of detail provided by Statistics Sweden, the need to construct consistent groupings over time, and in some cases ascertaining a sufficient number of observations for each category. Albeit somewhat subjective, the list of region groups approximately displays their respective degree of sociocultural similarity in ascending (albeit not necessarily linear) order. Consequently, on average, parents from the Nordic countries are presumed to experience less obstacles or effort to integrate linguistically, socioeconomically and in achieving intermarriage, compared to individuals from other European countries, who—in turn—do this with greater ease than foreign-born individuals from Asia and Africa. Similar to Jonsson and Rudolphi (2011), for individuals whose parents come from two separate countries, they are assigned the region of origin which is “closest” to Sweden. Our rationale for doing so is that this parent should be the one who on average provides the child with the most destination country-specific knowledge.

#### Years Since Migration

Parents’ years since migration is constructed as the parents’ number of years spent in Sweden at the time of the child’s birth. This is used as an approximate measure of integration and is expected to capture the accumulated effects of acculturation and structural assimilation. In households with two foreign-born parents reporting two unique years since migration values, we use the value for the foreign-born parent from the culturally “closest” region.

#### Intermarriage

Having intermarried parents is defined as when a second-generation child has one native-born and one foreign-born biological parent. The parents need not be married, however, but we believe that similar processes operate regardless of whether the child was born within or out of wedlock.

### Methods

The focus of this study is on estimating the impact of parents’ years spent in Sweden on the child’s educational performance, as measured by the standardized grade point average. One substantial empirical challenge is associated with the possibility that those who have a longer duration of stay may be fundamentally different from those having only stayed for a shorter period of time (Chiswick and Miller 1995), making it difficult to assess the causal effect of parents’ time spent in the country of destination. More specifically, those who wait longer to have a child after migration might be doing so due to their preferences for their children’s education, and thus not necessarily independently of their integration experiences. Moreover, parents who intermarry are also likely to be positively selected, representing another potential source of bias (Kalmijn 1998). Lastly, children’s educational outcomes are partly determined by ability, representing another major potential source of unobserved heterogeneity.

In attempting to overcome aforementioned sources of bias, the multivariate analyses rely on family (sibling) fixed effects models, an estimator that is well suited to overcome some of the sources of bias which may result from correlation between independent variables and the error term. By comparing outcomes between biological siblings, the influence of shared time-invariant characteristics, such as genetic traits (50% shared between siblings) and parents’ preferences toward their children’s education, is canceled out (Lawlor and Mishra 2009; Angrist and Pischke 2008). In this way, our approach is able to remove important potential sources of bias that would otherwise jeopardize the validity of our results. The primary drawback of this approach is, however, that it restricts our sample to families with more than one child born in Sweden, thereby introducing the question of the external validity of the results vis-à-vis families that only have singletons. We expect this to be a minor problem, as the proportion of families in our sample window with two or more children born in Sweden exceeds 70%, though we acknowledge their experience is may be quite different.

The empirical specification follows Eq. (1).

1 $$Y_{ij} = {\varvec{\upalpha}} + {\varvec{\upbeta}}X_{ij} + {\varvec{\Phi}}Z_{ij} + {\varvec{\upmu}}_{j} + {\varvec{\upvarepsilon}}_{ij}$$

Y_ij_ is a continuous variable, representing the grade point average of individual *i*, standardized by year of graduation, belonging to family *j.* This is modeled as a function of a vector of control variables, *X*_*ij*_, including birth order (1, 2, 3, 4 + ), sex and the assessment reform that took place from 1998 (Wikström 2006). The key parameter is represented by ${\varvec{\Phi}}$, estimated based on the parents’ years since migration (*Z*) when individual *i* in family *j* was born. The identification of all parameters relies on within-family variation (between siblings), and this not only pertains to observed characteristics, but also to unobserved characteristics, which implies that the influence of everything shared between siblings that could otherwise bias the estimates will be canceled out through the family fixed effect, parameter **μ**_*j*_ . Lastly, **ε**_*ij*_ is an individual specific error term.

The analysis is performed separately by parents’ region of origin and intermarriage status. This is necessary due to the time-invariant characteristics of these indicators, and it allows us to estimate the effect of parents’ years since migration separately by parent’s region of origin. Although we are unable to directly compare the effects of parents’ years since migration between generations or region of origin, we will discuss differences between these groups in terms of the direction of the effect of parents’ time in Sweden and only to a lesser extent compare the size of coefficients across models.

## Results

The demographic characteristics of the sample examined are described in Table 1. Consistent with the timing of certain migrant groups’ arrival in Sweden, the 2.0 and 2.5 generation children originating from European countries on average graduate a few years before their peers from Africa or Asia. Though the study design conditions on families with at least two children who graduate ninth grade during the observation window, the average family size across all groups exceeds two children. Therefore, this sample should represent the normative experience of families within these groups. Also consistent with previous research on Sweden showing that immigrants’ fertility behavior rapidly converges to that of natives (Andersson and Scott 2005), no major differences in the family size can be observed. Lastly, the column % *gen status* displays the proportion of individuals within each region of origin group that belongs to each generation. Consequently, among children with at least one parent originating from Africa, 54% belong to the 2.0 generation and 46% belong to the 2.5 generation. Similar to past findings (Dribe and Lundh 2011), there are large differences between the groups in implicit intermarriage rates, where over 70% of the children belonging to the Nordic and EU-15, North America and Oceania groups are children of intermarriage, with the opposite being the case for Lebanon/Turkey and Iraq.

**Table 1:** Demographic characteristics of sample

	Gen. status	zGPA	% Female	Ninth-grade year	Family size	% Gen status	*N*
Africa	2.0	− 0.17	50	2005.3	2.7	54	6002
	2.5	0.02	50	2002.1	2.5	46	5052
Iraq	2.0	− 0.17	49	2006.5	2.4	81	2042
	2.5	− 0.11	48	2005	2.5	19	475
Iran	2.0	0.21	50	2005.9	2.2	58	2596
	2.5	0.08	47	2004	2.5	42	1910
Lebanon/Turkey	2.0	− 0.37	49	2003	3.1	88	16,638
	2.5	− 0.20	48	2003.4	2.5	12	2267
Asia	2.0	0.08	48	2004.4	2.8	62	11,841
	2.5	0.08	48	2002.8	2.5	38	7397
South America	2.0	− 0.36	49	2003.9	2.4	44	4030
	2.5	0.01	48	2003.3	2.4	56	5077
Eastern Europe	2.0	− 0.01	49	2000.5	2.4	49	13,982
	2.5	0.07	49	2001	2.4	51	14,462
EU-15, N. America and Oceania	2.0	0.04	49	2000.2	2.4	18	7629
	2.5	0.16	49	2000.4	2.5	82	34,918
Nordic	2.0	− 0.22	48	1999	2.6	25	25,454
	2.5	− 0.06	49	2000.7	2.5	75	75,733

*Source*: SIP, 1989–2011

**Fig. 1:**
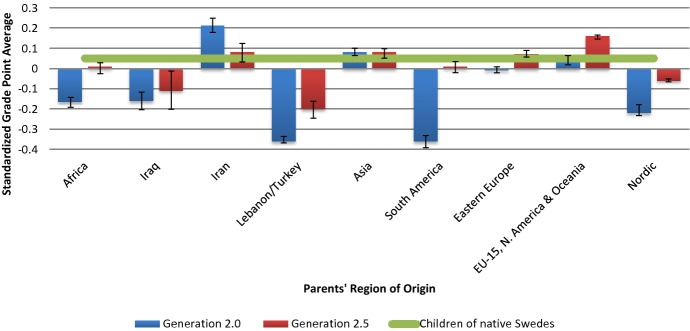
Average standardized grade point average by generation status and parents’ region of origin, 1989–2011. *Source:* SIP 1989–2011

The key independent variable for the study is parents’ years since migration, measured during the individual’s year of birth. In Fig. 1, the average value of parents’ years since migration by generation status and parents’ region of origin is displayed, along with bars which represent the within-family standard deviation, which represents the average birth spacing between children. Comparing the parents of 2.0 and 2.5 generation children, the foreign-born parent of the 2.5 generation has, on average, a longer duration of stay in Sweden before entering into parenthood than the 2.0 generation, particularly among parents from Iraq, Iran, Lebanon/Turkey, South America and the Nordic countries. Additionally, comparing between countries, we can see that parents who come from non-Western countries have a much shorter duration in Sweden before family formation, irrespective of intermarriage status, supporting what has been previously found on the heterogeneity of family formation behaviors in Sweden between countries of origin (Andersson 2004). Some of this difference, however, is influenced by the relatively older ages at which people from Western and non-Western groups tend to migrate, combined with a slightly earlier entry into parenthood (Andersson 2004). Conversely, those coming from countries with a history of labor migration tend to arrive at earlier ages and live in Sweden for a longer period of time prior to their first birth, a reason that may also contribute to their overall higher rate of intermarriage, similar to what has been found in the Netherlands (Kalmijn and van Tubergen 2006). Despite the variation in the average parents’ years since migration between region of origin groups and intermarriage status, the within-family standard deviation remains fairly uniform. Regardless of origin or generation, the average age gap between children is 2–3 years. The implication is that groups are displaying similar birth spacing practices, even if practicing different timing and total childbearing preferences. The combination of higher grades and greater parents’ years in Sweden observed for the 2.5 generation suggests there is an association between the two, but it may be confounded due to other, non-integration related, factors. This emphasizes the need to employ family fixed effect regression models in an effort to eliminate potential sources of confounders.

**Fig. 2:**
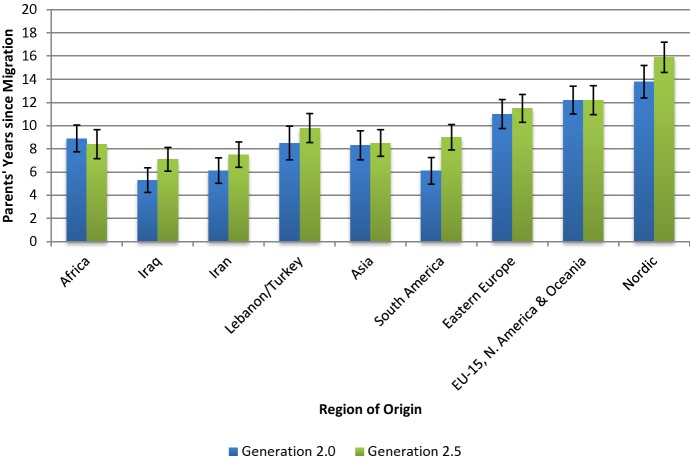
Average PYSM by parents’ region of origin, with within-family standard deviations, 1989–2011. *Source:* SIP 1989–2011

Figure 2 displays the average grade point average by generation status and parents’ region of origin group, along with confidence intervals. Addressing the overall relationship between parents’ region of origin and academic performance, the graph shows an expected pattern in which children of individuals from certain regions of origin perform substantially worse than children of native-born Swedes. Equally expected, the children of intermarried parents, the 2.5 generation, almost consistently outperform the 2.0 generation. For certain groups, most notably Africa and South America, a quite considerable GPA penalty among the 2.0 generation is among the 2.5 generation overturned into a performance almost on par with the children of native Swedes. An interesting exception is represented by children belonging to the Iranian and Asian groups, who outperform children of native Swedes in the 2.0 generation, but whose performance in the 2.5 generation is on par with the comparison group. Consequently, these figures largely reaffirm what has been found in studies both internationally and for Sweden; that children of foreign-born individuals generally perform worse compared to children of natives (Jonsson and Rudolphi 2011; Taguma et al. 2010). This supports the argument that 2.5 generation children are systematically different from the 2.0 generation (Kalmijn 2015, 2010; Ramakrishnan 2004), though it cannot address whether this difference is due to selection alone, integration achieved prior to intermarriage or whether it continues across the life course.

Table 2 presents results addressing hypotheses one and two, from the regression models on standardized grade point average, controlling for parents’ years since migration, sex, birth order and the assessment reform of 1998, stratified by parents’ region of origin. Furthermore, the models use family fixed effects to account for potential sources of selection bias that are shared at the sibling level. Since the examined hypotheses treat the children of foreign-born individuals as one group, regardless of whether they have one or two foreign-born parents, the estimates are for both the 2.0 and the 2.5 generation. The results show heterogeneity in terms of the size of the effect of the relationships between parents’ time in Sweden and their children’s grade performance depending on the parents region of origin, but with all point estimates displaying positive effects albeit not all being statistically significant. Consistent with Hypothesis 1, the positive post estimates indeed suggest that the foreign-born parents’ integration experience transmits to their children. While this is consistent with Hypothesis 1, some groups’ null effects, however, fail to provide comprehensive support for the hypothesis. Examining the estimates in more detail, it emerges that those that are statistically significant are all positive and that the largest effect is observed for the Lebanon/Turkey and the South America group, suggesting that the parent(s) having spent another year in Sweden at the time of their birth translates to a 3% of a standard deviation higher ninth-grade GPA. Considering the typical time between siblings’ births being 2–3 years, the de facto effects imply that the later born sibling will enjoy an approximately 6–9% of a standard deviation higher GPA. Interestingly, null effects are found for children with at least one parent born in Iraq or Iran.

**Table 2:** GLS regression with family fixed effects

Country of origin	PYSM	Constant	Observations	Family Obs.	*R* ^2^
Africa	0.02** (0.01)	− 0.36** (0.04)	12,870	5414	0.07
Iraq	0.01 (0.01)	− 0.11 (0.11)	3241	1443	0.05
Iran	0.01 (0.01)	0.05 (0.06)	5267	2419	0.05
Lebanon/Turkey	0.03** (0.00)	− 0.57** (0.03)	22,081	8329	0.05
Asia	0.01* (0.01)	− 0.05 (0.03)	17,267	7259	0.06
South America	0.03** (0.01)	− 0.39** (0.05)	9895	4446	0.05
Eastern Europe	0.01** (0.00)	− 0.18** (0.03)	29,603	13,348	0.07
EU-15, N. America and Oceania	0.02** (0.00)	− 0.11** (0.03)	41,642	18,221	0.07
Nordic	0.02** (0.00)	− 0.43** (0.03)	95,429	41,008	0.08

Dependent variable is grade point average, stratified by year, controlling for parent’s years since migration (PYSM), as well as sex, birth order (1, 2, 3 and 4 + ), and pre-/post-1998 assessment reform, stratified by parent’s region of origin

*Source*: SIP 1989–2011

Standard errors in parentheses, ^+^*p *< 0.10; *p < 0.05; **p < 0.01

Continuing to Hypothesis 2, the expectation is that the benefits of the foreign-born parents’ additional time spent in the country may be of limited value for the children of parents belonging to the groups at greatest risk of downward assimilation. Consequently, the lack of effect from parents’ years since migration among children with parents from Iran and Iraq emerges as in line with this hypothesis. Arguably, equally at risk of experiencing downward mobility is, however, parents from Africa, Asia and Lebanon/Turkey, whose integration experience clearly transmits to the subsequent generation. Indeed, siblings with parent(s) originating from Africa or Asia who were born 2 years apart are predicted to experience about a 4% of a standard deviation difference in GPA. These effects are similar in size to the effects for children of Nordic immigrant parents, illustrating the absence of diminishing returns to parental years since migration by the socioeconomic and cultural distance.

Remaining hypotheses redirect the focus to distinguish between the effects of parents’ time spent in the destination country among the 2.0 and the 2.5 generation, presented in Table 3. In line with the expectation that the acquisition of Sweden-specific skills is enhanced through intermarriage, Hypothesis 3a postulates that the effects of parents’ years since migration are greater for 2.5 generation than for 2.0 generation children. Comparing the effects within each region of origin group, the results fail to suggest this consistently being the case. More specifically, while the effects for the Lebanon/Turkey, South America, Eastern Europe and EU-15, North America and Oceania point toward larger effects among the intermarried, remaining five groups do not. Furthermore, regardless of whether the largest effects are observed among the 2.0 or 2.5 generation, the difference within each region of origin group in all but one case does not exceed 0.01, suggesting a de facto difference in GPA between siblings born 3 years apart amounting to 3% depending on whether the parents intermarried a native-born Swede.

**Table 3:** GLS regression with family fixed effects

Country of origin	Gen status	PYSM	Constant	Observations	Family obs.	*R* ^2^
Africa	2.0	0.02** (0.01)	− 0.45** (0.06)	6002	2445	0.07
	2.5	0.02** (0.01)	− 0.26** (0.06)	5052	2192	0.08
Iraq	2.0	− 0.00 (0.02)	− 0.19 (0.15)	2042	917	0.05
	2.5	0.01 (0.04)	− 0.17 (0.23)	475	207	0.16
Iran	2.0	0.01 (0.01)	0.02 (0.11)	2596	1240	0.05
	2.5	0.01 (0.02)	− 0.02 (0.10)	1910	839	0.04
Lebanon/Turkey	2.0	0.03** (0.00)	− 0.61** (0.03)	16,638	6137	0.05
	2.5	0.04** (0.01)	− 0.56** (0.10)	2267	981	0.04
Asia	2.0	0.02** (0.01)	− 0.23** (0.03)	11,841	4689	0.05
	2.5	0.00 (0.01)	− 0.05 (0.05)	7397	3235	0.07
South America	2.0	0.02* (0.01)	− 0.56** (0.06)	4030	1825	0.03
	2.5	0.03** (0.01)	− 0.23** (0.07)	5077	2270	0.07
Eastern Europe	2.0	0.01 (0.00)	− 0.20** (0.04)	13,982	6352	0.07
	2.5	0.01** (0.00)	− 0.18** (0.04)	14,462	6467	0.07
EU-15, N. America and Oceania	2.0	0.01 (0.01)	− 0.15** (0.07)	7629	3422	0.05
	2.5	0.02** (0.00)	− 0.10** (0.03)	34,918	15,215	0.08
Nordic	2.0	0.02** (0.00)	− 0.52** (0.04)	25,454	10,950	0.07
	2.5	0.01** (0.00)	− 0.38** (0.03)	75,733	32,531	0.08

Dependent variable is grade point average, standardized by year, controlling for parent’s years since migration (PYSM), sex, birth order (1, 2, 3 and 4 + ), and pre-/post-1998 assessment reform, stratified by parents’ region of origin and individual’s generation status

*Source*: SIP 1989–2011

Standard errors in parentheses, ^+^*p *< 0.10; *p < 0.05; **p < 0.01

In Hypothesis 3b, it is argued that the benefits associated with intermarriage increase with socioeconomic and cultural distance, as those most distant are argued to benefits disproportionally through the resources provided by a native spouse. Consistent with the hypothesis, the largest effects are indeed found for children of intermarried parents from Lebanon/Turkey (0.04) and South America (0.03), characterized by a considerably greater distance compared to parents from the Nordic countries (0.01), EU-15, North America and Oceania (0.02) and Eastern Europe (0.01). Again, the results, however, fail to consistently support the hypothesis, as the groups Asia, Iraq and Iran all display null effects from years since migration among the intermarried parents.

Lastly, we address the hypothesis that if the primary benefit among children of intermarriage to a native comes from the resources provided by the native-born spouse, the effect of years since migration will be greater among the 2.0 than among the 2.5 generation. Support for this hypothesis is only found for two very different groups: Nordic and Asia. It emerges most visibly for the Asia group, where a null effect of years since migration is observed among the 2.5 generation, to be compared to a 4–6% GPA difference between a typical sibling pair belonging to the 2.0 generation.

In order to ascertain the robustness of our results, a range of alternative model specifications have been estimated. Firstly, models have been estimated which stratify on whether it is the mother or father who has more years in Sweden as well as whether it is the mother or father who is foreign-born in a mixed marriage, neither of which yielded significant differences. Secondly, models have been estimated using the parent with the closest socioeconomic and cultural distance rather than the furthest in determining the parents’ region of origin. Thirdly, to explore possible nonlinearities in the effect of parents’ years since migration, we included a squared term, with little evidence suggesting that our preferred models are misspecified. Fourthly, models were stratified by highest parental education and Western/non-Western background, and parents’ years since migration had a significant and positive effect on standardized grade point average across all education categories. Fifthly, we tested an interaction between birth order and parents’ years since migration and conclude that the relationship is consistent across birth order. Furthermore, we stratified the models by sibship size, and, again, the results showed no indications that the individual’s number of siblings moderates the relationship between parent’s years since migration and educational performance. Finally, although parents’ time since migration is correlated with birth spacing, previous research at the population level in Sweden has found no effect between birth spacing and educational performance (Barclay and Kolk 2017) in the general population, implying that the effect of parent’s years since migration should be uninfluenced by birth spacing.^5^

## Conclusions and Discussion

A central tenet of assimilation theory is that time and generation in a host country will lead to increasing integration for individual migrants and their descendants. We investigate this by estimating the association between foreign-born individuals’ time in Sweden on their children’s educational performance, in terms of grade point average at age 16. We additionally assess whether these mechanisms of intergenerational transmission of integration also apply to children of intermarried parents. This study thus also contributes to the larger question of whether intermarriage serves only as a marker of completed integration among the first generation or whether its effects spill over to the next generation. The results tell a story of diverse experiences of the intergenerational transmission of integration, failing to fully conform to any of the outlined hypotheses. Thus, while certain groups experience consistent benefits from increased parental integration—for both the 2.0 and 2.5 generation—others fail to display either.

For the majority of the groups examined, the foreign-born parents’ time in Sweden does seem to be associated with advantages that are transmitted to their children, improving their educational performance. Previous research has postulated this to be the result of parents’ language proficiency, familiarity with the school system and the declining salience of cultural distance, as well coming to embrace and invest in living in the host country (Bleakley and Chin 2008; Casey and Dustmann 2008; Turney and Kao 2009; Smith et al. 2016). Notably, the outcome of interest here is a more general measure of academic performance, rather than focusing on language-related outcomes as in previous research (Smith et al. 2016).

Similarly, the continued positive effect of integration after intermarriage observed for some groups suggests that intermarriage may stand as point along a possible individual integration trajectory. That this occurs across origin groups with a range of backgrounds who arrive across a number of decades suggests this is not dependent on specific circumstances, but rather a general trend of integration. This finding supports the idea that 2.5 generation children exhibit better educational performance in part due to continued integration for the foreign-born parent which is then transmitted intergenerationally to their children. The implication is that the achievement of 2.5 generation children is neither only the result of positive selection into intermarriage among the foreign-born parent (Song 2009), nor is it purely the result of the one native-born parent.

There is a considerable degree of internal consistency between 2.0 and 2.5 generation children with respect to the effect of parents’ years since migration on children’s educational performance by generation status. However, whether the integration processes that affect these relationships are similar is debatable. Among intermarried couples with children, the native parent, presumably fluent in Swedish, brings familiarity with the school system and knowledge of institutions into their household, characteristics that households with two foreign-born parents need to acquire through integration. These differences in circumstances would lead us to expect the effect of parents’ years since migration on children’s performance to function differently for 2.0 and 2.5 generation individuals. Possible mechanisms include the expanded social network available to foreign-born spouses in mixed marriages (Goldstein 1999; Laumann et al. 1994), which might facilitate easier acculturation, as well as increased structural integration for their children (Kalmijn 2010). Although previous studies have documented individual integration continuing for the foreign-born intermarried parent (Dribe and Nystedt 2015; Tegunimataka 2017), the intergenerational transmission of this integration, in the presence of a native in the household, is perhaps unexpected.

The implications of the results for those groups who do not derive a benefit from parental time in Sweden, notably both 2.0 and 2.5 generation children whose parent(s) immigrated from Iraq and Iran, as well as the 2.0 generation from Eastern Europe, EU-15, North America and Oceania, and the Nordic countries, are less certain. These integration patterns fall outside of the predictions of assimilation theory. This lack of relationship could be the result of parents not integrating with time in Sweden or parents’ experiences not transmitting across generations. This result is surprising for the 2.0 generation from EU-15, North America and Oceania, as they do not fit the model of those at risk of downward assimilation. It is especially interesting in light of the significant relationship observed for the 2.5 generation and raises questions as to why this relationship would be observed for them, but not the 2.0 generation. Also, Iranian second-generation children have grade point averages roughly on par with native Swedes. This is possibly the result of a positive selection among Iranian immigrants to Sweden, who arrived with higher levels of education than other refugees (Haberfeld and Lundh 2014; Aradhya et al. 2016). Second-generation Iranian children also seem unlikely to be at risk of downward assimilation despite the absence of a relationship between parents’ years since migration and children’s grade performance. Instead, this group may be performing so highly already that parents’ additional time in Sweden does not have the effect that is observed elsewhere. In comparison, Iraqi second-generation children have a lower mean standardized grade point average of −0.12 and display no real improvement with parents’ additional time in Sweden. This lack of relationship among the Iraqi children may be the clearest example of downward assimilation, given their very disadvantaged position in the Swedish labor market (Bevelander 1999, 2011).

Previous studies looking at the intergenerational transmission of integration have largely focused on language performance and proficiency (Bleakley and Chin 2008; Casey and Dustmann 2008) or non-academic social integration (Martinovic et al. 2009). This study expands beyond this and finds, overall, a positive relationship between parental years in Sweden and children’s overall grade point average. The implication is that parental time in Sweden provides a more general benefit, even for courses which are less reliant on language, that has not been found in previous research (Smith et al. 2016). The positive effect of parental time on their children’s academic performance suggests that assimilation is the overarching trend and that the majority of the foreign-born population is using their time in Sweden to integrate. This is transferred to their children who are able to academically benefit from their parents’ time in Sweden. This development is perhaps most notable among groups that have low achievement levels, such as the Africans, Turks and Lebanese and South Americans (Taguma et al. 2010). These groups are at risk of downward assimilation, due to their low levels of human capital and non-European background, but they do appear to be assimilating with positive intergenerational consequences.

Though assimilation appears to be the dominant trend, certain groups do not enjoy the same benefit, most notably those with an Iraqi or Iranian background. Among these groups, the hypothesized effects of assimilation are not found and perhaps indicate a process of stagnation or downward assimilation. The children of Iranian immigrants, however, might be a special case due to higher general academic performance than other groups (Haberfeld and Lundh 2014; Aradhya et al. 2016). Compared to Iranian migrants, Iraqi immigrants arrived with lower levels of human capital and have experienced limited upward social mobility (Bevelander 1999, 2011). The neutral relationship between parental time and children’s grade point average observed for Iraqis, along with relatively low average standardized grade point average for the group, suggests that they are the group most at risk of downward assimilation.

The overall positive effect of parents’ years since migration on the educational performance of the 2.5 generation is a notable finding, particularly since previous research has focused on the role of parents’ linguistic acculturation on child’s proficiency and class performance (Bleakley and Chin 2008; Casey and Dustmann 2008; Smith et al. 2016). Among children of intermarried parents, it might be anticipated that the influence of the integration of the foreign-born parent is limited. Additionally, if intermarriage is an outcome of positive integration, then the effect of parents’ additional time in Sweden should tend toward zero, since integration should have been fairly complete at entry into parenthood. Instead, the results for several groups suggest that the foreign-born parent’s integration continues past this event. In this light, this study supports a developing line of research that is suggesting that intermarriage is more than an outcome of integration (Dribe and Nystedt 2015; Furtado and Song 2015; Tegunimataka 2017). Instead, intermarriage can act as a vehicle for continued integration with intergenerational consequences, possibly as a result of increased inclusion in native-dominated social networks and easier access to cultural and structural information. That these findings are present when employing a family fixed effect approach suggests that continued integration plays a part in the 2.5 generation outperforming the 2.0 generation, indicating that this is due to more than the consequences of selection into intermarriage alone (Furtado 2009).

Although we believe our approach provides valuable insights into the question of whether parents’ integration, as measured by time in a host country, can have intergenerational consequences in terms of their children’s educational performance, certain limitations exist. For one, as previously mentioned, the external validity to families with one child is unattainable using our sibling-based approach. Although this group represents a minority of families among the cohorts examined, it is nonetheless a weakness. Also, although grade point averages across all subjects is the most comprehensive indicator of academic achievement at the end of ninth grade, it is important to recognize that grades are influenced by other factors than knowledge of the topics studied, including classroom behavior and teacher differences in grading routines. Finally, while the method employed in this paper offers important advantages in terms of canceling out the influence of a range of factors which would be likely to bias the results, it is not able to account for unobserved factors at the family level that vary over time, including the increasing experience of being a parent.

In employing a family fixed effect approach, this study provides a more refined look at the potential effect of parents’ time in Sweden on children’s educational performance. This is the result of measuring only the time-varying features that change within the family. Many of these features fall under the umbrella category of dimensions of integration, e.g., increased language proficiency, knowledge of school systems, which we are attempting to capture with our general measure of integration. This paper establishes a general effect of parents’ time in Sweden on children’s educational performance, but does not explore the mechanisms behind the integration process. A political implication of this finding is that it is important to provide immigrants an opportunity to begin the integration process as quickly as possible, particularly in light of the recent refugee crisis in Europe, in which some countries have taken certain measures to make their countries unattractive to refugees and more difficult to integrate into, such as cutting integration benefits, restricting family reunification and placing refugees in rural locations (Bilefsky 2016; Delman 2016), which could have negative immediate and long-term implications for these groups. Future research into the topic of intergenerational transmission could expand beyond this general measure and explore more specifically the role different components of integration may have in this relationship.

## Appendix

See Tables 4, 5, and 6.

**Table 4:** Region of origin grouping

Country of origin group	Nordic	EU-15, N. America and Oceania	Eastern Europe	South America	Asia	Lebanon/Turkey	Iran	Iraq	Africa
Countries of origin included	Denmark	Switzerland	Bosnia–Herzegovina	Chile	Thailand	Lebanon	Iran	Iraq	Somalia
	Norway	Germany	Serbia	Colombia	China	Turkey			Ethiopia
	Finland	United Kingdom	Montenegro	Peru	Syria				Eritrea
	Iceland	Greece	Czechoslovakia	Brazil	India				Morocco
		Netherlands	Ukraine	Bolivia	Vietnam				Tunisia
		France	Poland		Afghanistan				Gambia
		Italy	Romania		South Korea				Egypt
		Spain	Hungary		Pakistan				Uganda
		Austria	Estonia		Philippines				
		Australia	Lithuania		Sri Lanka				
		USA	Bulgaria		Bangladesh				
		Canada	Croatia		Palestine				
			Latvia						
			Soviet Union						
			Russia						

The table includes countries of birth represented by at least 3000 individuals in Sweden in 2010. Listed countries of birth represent 93% of the foreign-born population in Sweden

**Table 5:** Full regression result from Table 2

	Africa	Iraq	Iran	Lebanon/Turkey	Asia	South America	Eastern Europe	EU-15, N. America and Oceana	Nordic
PYSM	0.02** (0.01)	0.01 (0.01)	0.01 (0.01)	0.03** (0.00)	0.01* (0.01)	0.03** (0.01)	0.01** (0.00)	0.02** (0.00)	0.02** (0.00)
Sex (ref: male)									
Female	0.39** (0.02)	0.29** (0.04)	0.27** (0.03)	0.33** (0.01)	0.33** (0.01)	0.30** (0.02)	0.34** (0.01)	0.34** (0.01)	0.39** (0.01)
Sibling order (ref: oldest)									
Sibling 2	− 0.10** (0.02)	− 0.13** (0.05)	− 0.13** (0.04)	− 0.11** (0.02)	− 0.10** (0.02)	− 0.18** (0.03)	− 0.15** (0.01)	− 0.16** (0.01)	− 0.16** (0.01)
Sibling 3	− 0.24** (0.04)	− 0.15 + (0.09)	− 0.14* (0.07)	− 0.19** (0.03)	− 0.15** (0.03)	− 0.29** (0.05)	− 0.25** (0.03)	− 0.29** (0.02)	− 0.28** (0.02)
Sibling 4 or more	− 0.35** (0.07)	− 0.13 (0.16)	− 0.32* (0.13)	− 0.25** (0.04)	− 0.22** (0.05)	− 0.40** (0.09)	− 0.33** (0.05)	− 0.37** (0.04)	− 0.38** (0.02)
Reform	− 0.04 (0.04)	− 0.12 (0.11)	− 0.01 (0.06)	− 0.05* (0.03)	0.01 (0.03)	− 0.03 (0.04)	0.02 (0.02)	− 0.01 (0.02)	0.01 (0.01)
Constant	− 0.36** (0.04)	− 0.11 (0.11)	0.05 (0.06)	− 0.57** (0.03)	− 0.05 (0.03)	− 0.39** (0.05)	− 0.18** (0.03)	− 0.11** (0.03)	− 0.43** (0.03)
Individual Obs.	12,870	3241	5267	22,081	17,267	9895	29,603	41,642	95,429
Family Obs.	5414	1443	2419	8329	7259	4446	13,348	18,221	41,008
*R* ^2^	0.07	0.05	0.05	0.05	0.06	0.05	0.07	0.07	0.08

Standard errors in parentheses, ^+^*p *< 0.10; **p *< 0.05; ***p *< 0.01

**Table 6:** Full regression results from Table 3

	Africa		Iraq		Iran		Lebanon/Turkey		Asia	
	2.0	2.5	2.0	2.5	2.0	2.5	2.0	2.5	2.0	2.5
PYSM	0.02**	0.02**	− 0.00	0.01	0.01	0.01	0.03**	0.04**	0.02**	0.00
	(0.01)	(0.01)	(0.02)	(0.04)	(0.01)	(0.02)	(0.00)	(0.01)	(0.01)	(0.01)
Sex (ref: male)										
Female	0.38**	0.42**	0.31**	0.43**	0.28**	0.25**	0.34**	0.30**	0.31**	0.35**
	(0.02)	(0.03)	(0.05)	(0.09)	(0.04)	(0.05)	(0.02)	(0.04)	(0.02)	(0.02)
Sibling order (ref: oldest)										
Sibling 2	− 0.08**	− 0.15**	− 0.10+	− 0.26*	− 0.13*	− 0.14*	− 0.11**	− 0.13*	− 0.07**	− 0.13**
	(0.03)	(0.03)	(0.06)	(0.12)	(0.05)	(0.06)	(0.02)	(0.05)	(0.02)	(0.03)
Sibling 3	− 0.21**	− 0.33**	0.05	− 0.54*	− 0.17	− 0.16	− 0.17**	− 0.34**	− 0.17**	− 0.19**
	(0.05)	(0.07)	(0.11)	(0.24)	(0.11)	(0.12)	(0.03)	(0.10)	(0.04)	(0.06)
Sibling 4 or more	− 0.28**	− 0.57**	0.03	− 0.59	− 1.01**	− 0.32	− 0.25**	− 0.48**	− 0.19**	− 0.35**
	(0.09)	(0.11)	(0.19)	(0.43)	(0.29)	(0.20)	(0.05)	(0.16)	(0.06)	(0.09)
1998 reform	0.00	− 0.02	− 0.09	− 0.04	0.03	0.03	− 0.07*	− 0.04	− 0.01	0.02
	(0.06)	(0.06)	(0.15)	(0.23)	(0.11)	(0.09)	(0.03)	(0.10)	(0.03)	(0.04)
Constant	− 0.47**	− 0.26**	− 0.29**	− 0.19	0.06	− 0.02	− 0.62**	− 0.56**	− 0.17**	− 0.05
	(0.06)	(0.06)	(0.06)	(0.20)	(0.06)	(0.10)	(0.03)	(0.10)	(0.03)	(0.05)
Individual obs.	6002	5052	2042	475	2596	1910	16,638	2267	11,841	7397
Family Obs.	2445	2192	917	207	1240	839	981	3235	1825	3235
*R* ^2^	0.07	0.08	0.05	0.16	0.05	0.04	0.05	0.04	0.05	0.07

	South America		Eastern Europe		EU-15, N. America and Oceana		Nordic	
	2.0	2.5	2.0	2.5	2.0	2.5	2.0	2.5
PYSM	0.02*	0.03**	0.01	0.01**	0.01	0.02**	0.02**	0.01**
	(0.01)	(0.01)	(0.00)	(0.00)	(0.01)	(0.00)	(0.00)	(0.00)
Sex (ref: male)								
Female	0.24**	0.34**	0.34**	0.34**	0.30**	0.34**	0.40**	0.38**
	(0.03)	(0.03)	(0.02)	(0.02)	(0.02)	(0.01)	(0.01)	(0.01)
Sibling order (ref: oldest)								
Sibling 2	− 0.13**	− 0.20**	− 0.12**	− 0.17**	− 0.14**	− 0.17**	− 0.15**	− 0.16**
	(0.04)	(0.03)	(0.02)	(0.02)	(0.03)	(0.01)	(0.02)	(0.01)
Sibling 3	− 0.20*	− 0.35**	− 0.23**	− 0.28**	− 0.18**	− 0.32**	− 0.25**	− 0.28**
	(0.08)	(0.07)	(0.04)	(0.04)	(0.05)	(0.02)	(0.03)	(0.02)
Sibling 4 or more	− 0.29+	− 0.41**	− 0.33**	− 0.36**	− 0.09	− 0.41**	− 0.32**	− 0.39**
	(0.15)	(0.12)	(0.07)	(0.07)	(0.10)	(0.04)	(0.05)	(0.03)
1998 reform	0.00	− 0.03	0.02	0.03	− 0.04	0.01	− 0.04	0.02
	(0.06)	(0.06)	(0.03)	(0.03)	(0.05)	(0.01)	(0.02)	(0.01)
Constant	− 0.56**	− 0.25**	− 0.21**	− 0.17**	− 0.16*	− 0.11**	− 0.49**	− 0.39**
	(0.05)	(0.07)	(0.04)	(0.04)	(0.07)	(0.03)	(0.04)	(0.03)
Individual observations	4030	5077	13,982	14,462	7629	34,918	25,454	75,733
Family obs.	1825	2270	6352	6467	3422	15,215	10,950	32,531
*R* ^2^	0.03	0.07	0.07	0.07	0.05	0.08	0.07	0.08

*Source*: SIP 1989–2011

Standard errors in parentheses, ^+^*p *< 0.10; **p *< 0.05; ***p *< 0.01

## Footnotes

1 Assimilation is used in this study in reference to past theories or concepts where appropriate, while integration is more generally used to describe the changes immigrants and their families undergo in response to migration as it more accurately represents the multidimensionality of immigrants’ experience, particularly in a country with a multicultural perspective such as in Sweden.

2 From the 2010 academic year.

3 The EU-15 is preferred over the EU-27 since immigrant parents must have migrated to Sweden prior to 2004 when this expansion took place. Many of these countries had previously been in the Eastern European sphere of influence and are categorized accordingly.

4 A detailed table of the country of birth grouping is provided in Table 4, “Appendix.”

5 Results available upon request.
